# Integration of tumour sequencing and case–control data to assess pathogenicity of *RAD51C* missense variants in familial breast cancer

**DOI:** 10.1038/s41523-021-00373-y

**Published:** 2022-01-17

**Authors:** Belle W. X. Lim, Na Li, Simone M. Rowley, Ella R. Thompson, Simone McInerny, Magnus Zethoven, Rodney J. Scott, Lisa Devereux, Erica K. Sloan, Paul A. James, Ian G. Campbell

**Affiliations:** 1 Cancer Genetics Laboratory, Peter MacCallum Cancer Centre, Melbourne, VIC Australia; 2 Drug Delivery Biology, Monash Institute of Pharmaceutical Sciences, Monash University, Melbourne, VIC Australia; 3 Sir Peter MacCallum Department of Oncology, The University of Melbourne, Melbourne, VIC Australia; 4 Parkville Familial Cancer Centre, Peter MacCallum Cancer Centre and Royal Melbourne Hospital, Melbourne, VIC Australia; 5 Department of Pathology, Peter MacCallum Cancer Centre, Melbourne, VIC Australia; 6 Bioinformatics Consulting Core, Peter MacCallum Cancer Centre, Melbourne, VIC Australia; 7 Discipline of Medical Genetics and Centre for Information-Based Medicine, The University of Newcastle and Hunter Medical Research Institute, Newcastle, NSW Australia; 8 Division of Molecular Medicine, Pathology North, Newcastle, NSW Australia; 9 Lifepool, Peter MacCallum Cancer Centre, Melbourne, VIC Australia; 10 Peter MacCallum Cancer Centre Division of Surgery, Melbourne, VIC Australia; 11 Cancer Genomics Program, Peter MacCallum Cancer Centre, Melbourne, VIC Australia

**Keywords:** Breast cancer, Cancer genetics

## Abstract

While protein-truncating variants in *RAD51C* have been shown to predispose to triple-negative (TN) breast cancer (BC) and ovarian cancer, little is known about the pathogenicity of missense (MS) variants. The frequency of rare *RAD51C* MS variants was assessed in the BEACCON study of 5734 familial BC cases and 14,382 population controls, and findings were integrated with tumour sequencing data from 21 cases carrying a candidate variant. Collectively, a significant enrichment of rare MS variants was detected in cases (MAF < 0.001, OR 1.57, 95% CI 1.00–2.44, *p* = 0.05), particularly for variants with a REVEL score >0.5 (OR 3.95, 95% CI 1.40–12.01, *p* = 0.006). Sequencing of 21 tumours from 20 heterozygous and 1 homozygous carriers of nine candidate MS variants identified four cases with biallelic inactivation through loss of the wild-type allele, while six lost the variant allele and ten that remained heterozygous. Biallelic loss of the wild-type alleles corresponded strongly with ER- and TN breast tumours, high homologous recombination deficiency scores and mutational signature 3. Using this approach, the p.Gly264Ser variant, which was previously suspected to be pathogenic based on small case–control analyses and loss of activity in in vitro functional assays, was shown to be benign with similar prevalence in cases and controls and seven out of eight tumours showing no biallelic inactivation or characteristic mutational signature. Conversely, evaluation of case–control findings and tumour sequencing data identified p.Ile144Thr, p.Arg212His, p.Gln143Arg and p.Gly114Arg as variants warranting further investigation.

## Introduction

Protein-truncating variants in *RAD51C* predispose to high-grade serous ovarian cancer (HGSOC) and triple-negative (TN) breast cancer (BC), and when these cancers occur in carriers of truncating variants they exhibit biallelic inactivation^1–3^. Few studies have investigated whether missense (MS) variants of *RAD51C* exert similar penetrance as protein-truncating variants. BC case–control studies to date have identified potentially predisposing *RAD51C* MS variants, such as p.Gly264Ser^4–6^, p.Gln143Arg^7,8^ and pArg258His^9,10^, while target protein and cellular assays have suggested functional impact and pathogenicity of variants including p.Cys135Tyr and p.Gly264Ser^5,8,9^. However, the sample sizes in these studies were small, with conflicting evidence presented for many variants. To address this, we analysed data from the BEACCON study of 5734 familial BC cases and 14,382 population controls^2^ for rare *RAD51C* MS variants (MAF < 0.005). To further investigate the potential pathogenicity of candidate variants, we exploited the fact that *RAD51C* appears to conform to Knudson’s “two-hit” hypothesis, and performed tumour sequencing from variant carriers to assess for biallelic inactivation and associated homologous recombination deficiency (HRD). We have previously demonstrated the utility of this reproach for *RAD51C* loss of function (LoF) variants by revealing the presence of biallelic inactivation in the form of loss of heterozygosity (LOH) in TN BCs that was also associated with high HRD scores and mutational signature 3^1^. In this study, case–control analysis data were combined with tumour sequencing, in silico prediction tools, and pedigree segregation to assess the pathogenicity of *RAD51C* MS variants.

## Results

### Likely pathogenic variants were enriched in the case cohort

A total of 51 unique rare MS variants (MAF < 0.005) were detected in 65 cases (1.13%) and 134 controls (0.91%) (OR 1.22, 95% CI 0.89–1.65, *p* = 0.21) (Table 1). Several parameters were used to enrich potentially pathogenic variants including population frequency, variant location in known functional domains, in silico pathogenicity prediction and tumour phenotype. Consistent with the hypothesis that rare variants are more likely to be deleterious^11^, a reduction of the population frequency threshold resulted in increasing odds ratios that reached statistical significance at MAF < 0.0001 (OR 1.87, 95% CI 1.14–3.03, *p* = 0.01). Similarly, higher CADD and REVEL score thresholds that should enrich for pathogenic variants were associated with higher odds ratios, especially for a REVEL score of >0.5 (OR 3.95, 95% CI 1.40–12.01, *p* = 0.006). The majority of variants exist at a very low population frequency, with only two variants (p.Val169Ala and p.Gly264Ser) that had REVEL scores >0.3 and CADD scores >25 reaching a MAF of >0.0001. Two overlapping functional domains are present in the N-terminal third of RAD51C protein (Holliday junction activity: amino acids 1–126; Interaction with RAD51B, RAD51D and XRCC3: amino acids 79–136) and significant enrichment of MS variants in cases was observed in the interaction domain (OR 10.04, 95% CI 0.99–494.1, *p* = 0.03), although the number of variants was small (*n* = 5), resulting in a wide confidence interval. Three very rare variants in four individuals were detected within the Walker B domain (two cases and two controls), while no variants were detected within the Walker A domain.

**Table 1:** Frequencies of *RAD51C* MS variants in case and control cohorts according to different filtering criteria to enrich for likely pathogenic variants.

	Groups	Carrier frequency		Sample size		*p* value	OR (95% CI)
		Case (%)	Control (%)	Case	Control		
Rarity	MAF < 0.005	65 (1.13)	134 (0.91)	5734	14,382	0.21	1.22 (0.89–1.65)
	MAF < 0.001	35 (0.61)	56 (0.38)			0.05	1.57 (1.00–2.44)
	MAF < 0.0001	32 (0.56)	43 (0.29)			0.01	1.87 (1.14–3.03)
In silico prediction	CADD > 20	59 (1.03)	125 (0.85)			0.29	1.19 (0.85–1.63)
	CADD > 25	17 (0.30)	22 (0.15)			0.05	1.94 (0.97–3.83)
	REVEL > 0.3	17 (0.30)	19 (0.13)			0.02	2.25 (1.10–4.57)
	REVEL > 0.5	11 (0.19)	7 (0.05)			0.006	3.95 (1.40–12.01)
Functional domain	Interaction domain	4 (0.070)	1 (0.01)			0.03	10.04 (0.99–493.1)
	Holliday domain	6 (0.10)	7 (0.05)			0.21	2.15 (0.60–7.48)
	Walker domains	2 (0.03)	2 (0.01)			0.31	2.21 (0.61–7.67)
Hormone receptor subtype	ER-positive	23 (1.04)	134 (0.91)	2209		0.64	1.12 (0.68–1.75)
	ER-negative	20 (1.58)		1262		0.04	1.70 (1.00–2.74)
	HER2-positive	7 (1.21)		579		0.51	1.30 (0.51–2.77)
	HER2-negative	29 (1.20)		2426		0.22	1.28 (0.83–1.93)
	TN	13 (1.49)		871		0.11	1.60 (0.83–2.85)
	Non-TN	23 (1.08)		2125		0.47	1.16 (0.71–1.82)

*MAF* minor allele frequency, *CADD* Combined Annotation-Dependent Depletion score, *REVEL* rare exome variant ensemble learner score, *ER* estrogen receptor, *HER2* human epidermal growth factor receptor 2, *TN* triple-negative.

Subgroup analysis based on hormone receptor status was carried out on case subjects where detailed pathology data were available from the Variant in Practice (ViP) study (*n* = 3645). Consistent with previous findings for *RAD51C* LoF carriers, rare MS variants were significantly enriched in the ER-negative BC subgroup (OR 1.70, 95% CI 1.00–2.74, *p* = 0.04), with a similar but non-significant trend in TN BC cases (OR 1.60, 95% CI 0.83–2.85, *p* = 0.11).

**Fig. 1:**
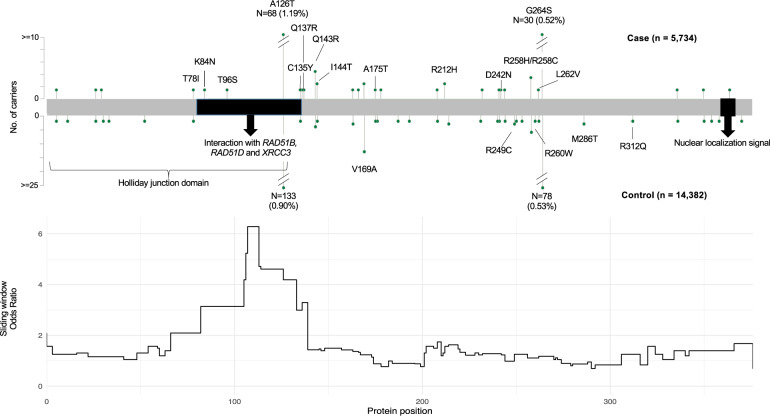
The location and frequency of *RAD51C* MS variants detected in cases (*n* = 5734) and controls (*n* = 14,382), and case–control odds ratios in position-based analysis. Key variants are marked with protein change and variants of interest are pointed with arrows. Holliday junction domain includes protein position 1–126, domain interacting with RAD51B, RAD51D and XRCC3 include protein position 79–136. p.Ala126Thr, an accepted benign variant, is included as a reference in this figure but not in the analysis. Note the *y*-axis scale is different for cases and controls, accounting for the control cohort being more than twice as larger than the case cohort.

The distribution and frequency of rare MS variants across *RAD51C* in the 5734 cases and 14,382 controls were summarised in Fig. 1. While rare MS variants were distributed across the entire gene, cases showed higher frequencies in the initial half of the gene. The position-based odds ratio analysis showed a higher case–control odds ratio for variants located between amino acid positions 82 and 136, coinciding with the interaction domain.

### Variants of interest detected in cases and controls

Details of the rare *RAD51C* MS variants identified in this study including case–control numbers, in silico pathogenicity prediction and literature evidence were summarised in Supplementary Table 1. Also included is the reference variant p.Ala126Thr (MAF = 0.0054), a generally accepted benign variant. All of the variants were very rare (MAF ≤ 0.0001), with the exception of p.Gly264Ser (MAF = 0.0034). Despite the large sample size, most variants were detected in less than three subjects; therefore, the frequencies alone were not adequately powered to confirm or refute pathogenicity. The data did, however, suggest that p.Ala126Thr and p.Gly264Ser do not represent high-penetrance alleles. p.Ala126Thr was detected in 68 (1.19%) cases and 133 (0.9%) controls (OR 1.29, *p* = 0.10), close to the allele frequency reported in gnomAD database. Similarly, p.Gly264Ser was detected with equal frequencies in cases (*n* = 30, 0.52%) and controls (*n* = 78, 0.53%) (OR 0.96, *p* = 0.92).

### Sequencing of tumours from MS variant carriers

Twenty invasive breast tumours and one HGSOC from 21 cases were analysed using whole-exome sequencing (*n* = 5), Sanger sequencing (*n* = 2), and/or a targeted sequencing gene panel that included all exons and intron boundaries of *RAD51C* and other common BC somatically mutated genes (*n* = 14) (Table 2). These tumours were from cases that carried one of nine heterozygous candidate variants (p.Gly264Ser, p.Lys84Asn, p.Gln143Arg, p.Ile144Thr, p.Arg212His, p.Asp242Asn, p.Ile244Val, p.Arg258His and p.Leu262Val) as well as one homozygous p.Gly264Ser carrier. Of the 20 germline heterozygous carrying tumours, four were found to harbour a second hit through loss of the wild-type allele (LOH). However, another five had lost the mutant allele while eleven others remained heterozygous. On further investigation, none of the heterozygous cases showed evidence of promoter hyper-methylation or somatic point mutations in *RAD51C*. Of the eight tumours from heterozygous carriers of the p.Gly264Ser allele, only four showed copy number loss with three of these involving loss of the variant allele. Importantly, both the p.Gly264Ser homozygous carrier and the case with loss of the wild-type allele had HRD scores below those indicative of loss of homologous recombination function^12^.

**Table 2:** Molecular analysis of 21 tumours from *RAD51C* MS variant carriers.

Sample	Variant	Hormone receptor/HER2 status	Allele status	HRD score	Promoter hyper-methylation	*TP53* somatic mutation	Driver genes somatic mutations
1	p.Gly264Ser	TN	Germline homozygous	37	N/A	Mutated	None
2		TN	Wild-type loss	15	No	Mutated	*MAP3K1, RB1*
3		TN	Variant loss	122	No	Mutated	*NOTCH1, NOTCH2*
4^a^		TN	Variant loss	N/A	No	N/A	N/A
5		TN	Heterozygous	39	N/A	Mutated	*GATA3*
6^a^		ER–/HER2–	Variant loss	43	No	Mutated	*NOTCH2*
7		ER+/HER2–	Heterozygous	10	N/A	Wild-type	None
8		ER+/HER2–	Heterozygous	29	No	Wild-type	*NOTCH1*
9		ER+/HER2+	Heterozygous	28	No	Mutated	None
10^b^	p.Lys84Asn	ER–/HER2+	Heterozygous	12	N/A	Wild-type	None
11	p.Glu143Arg	TN	Variant loss	67	No	Mutated	*PTEN*
10^b^		ER–/HER2+	Heterozygous	12	N/A	Wild-type	None
12		ER+/HER2–	Heterozygous	6	No	Wild-type	*NOTCH2*
13		ER+/HER2–	Heterozygous	N/A	No	N/A	N/A
14	p.Ile144Thr	TN	Wild-type loss	78	No	Mutated	*NOTCH2*
15		ER+/HER2–	Heterozygous	18	No	Wild-type	None
16	p.Arg212His	ER–/HER2+	Wild-type loss	47	No	Mutated	*NOTCH2*, *KMT2C*, *NOTCH1*
17		ER+/HER2–	Heterozygous	10	No	Wild-type	*MAP3K1*
18	p.Asp242Asn	ER+/HER2+	Variant loss	49	No	Wild-type	None
19	p.Ile244Val	TN	Variant loss	78	No	Mutated	None
20	p.Arg258His	OvCa	Wild-type loss	70	No	Mutated	None
21	p.Leu262Val	ER+/HER2–	Heterozygous	28	No	Wild-type	*NOTCH2*

All samples were sequenced using a 485-gene targeted panel, with the exception of samples 1, 2, 14, 16 and 20 with whole-exome and samples 4 and 13 with exon-specific Sanger sequencing.

*HRD* homologous recombination deficiency.

^a^Carriers are first-degree related.

^b^Subject carries two *RAD51C* MS variants.

Loss of the wild-type allele was identified in two TN tumours carrying p.Ile144Thr and p.Arg212His variants, respectively, with both showing high HRD scores, while ER-positive tumours carrying these variants remained heterozygous. An ovarian tumour carrying p.Arg258His also showed LOH and had a high HRD score of 70. Of the four tumours sequenced that carried the p.Glu143Arg variant, the one TN case was found to have lost the variant allele, while the one ER-negative and two ER-positive tumours remained heterozygous. All three tumours carrying a germline p.Leu262Val, p.Ile244Val or p.Asp242Asn variant were also shown to remain heterozygous. Across all tumours, HRD scores were generally higher among TN BC and those that had locus-specific LOH (Supplementary Fig. 1) but the differences were more pronounced in tumours carrying candidate variants compared to carriers of p.Gly264Ser. Apart from *RAD51C*, none of the high HRD tumours harboured biallelic loss of other known HRD drivers, such as *BRCA1* or *BRCA2*.

Previous studies have shown that breast tumours from individuals carrying an LoF mutation in *RAD51C* accompanied with loss of the wild-type allele were associated with single base substitution mutational signature 3^1,13^. Therefore, the presence of mutational signature 3 was assessed for tumours from carriers of candidate MS variants. Paired tumour-normal whole-exome sequencing was carried out for five tumours from subjects 1, 2, 14, 16 and 20, that were germline homozygous for the variant or showed loss of the wild-type allele. Case 14 was a TN tumour carrying p.Ile144Thr that showed both a high proportion of signature 3 and a high HRD score (HRD = 78). In contrast, the ER-negative/HER2-positive tumour from subject 16 did not show signature 3. The HGSOC carrying the p.Arg258His variant (subject 20) showed a large proportion of signature 3. In the two carriers of p.Gly264Ser, the TN tumour from the germline homozygous carrier (subject 1) had few somatic mutations and showed only a small proportion of signature 3. The second case was also a TN tumour with loss of the wild-type allele and showed a high proportion of signature 12 that has no known aetiology. The remaining tumours that were sequenced with a large exome panel were combined in the analysis to achieve a minimum input of 50 somatic mutations (Supplementary Fig. 2). The contribution of signature 3 in tumours carrying a VUS was similar to the tumours carrying the p.Gly264Ser variant. When stratified based on tumour pathology, TN tumours as a group had a higher proportion of signature 3 and higher HRD scores than ER-positive tumours, but there was no clear distinction between VUS and benign variant carriers. The extent of LOH in chromosome 17q varied across tumours with high HRD scores, with many showing hemizygous loss across *BRCA1*, *RAD51C* and *RAD51D* (Supplementary Table 2). However, no biallelic loss was observed in other known HR driver genes including *BRCA1* and *BRCA2*, excluding the possibility that HRD may be caused by other known factors.

### Pedigree segregation of MS variant carriers

Nine additional family members from seven families (representing three different variants), were available for segregation analysis of the germline variant detected in the index case (Supplementary Fig. 3). In four families carrying the p.Gly264Ser variant, the variant was found to be present in two affected first-degree relatives (FDR) (ER+ BC 43, BC 50), but absent in two affected second-degree relatives (ER+ BC 38, lobular ER+ BC 56) of the respective index cases. In another family, the variant p.Gln143Arg was present in FDR diagnosed with TN BC (age 55), ER-positive BC (age 72) and HGSOC (age 74), while none of the three unaffected FDR tested carried the variant. Finally, the daughter of an index case carrying the p.Gln137Arg variant remained unaffected but is currently only 35 years old.

## Discussion

Germline protein-truncating variants in *RAD51C* are known to be associated with predisposition to developing HGSOC and TN BC^1,5,14^ but whether there are MS variants of equivalent penetrance is unclear. Data from the BEACCON study have demonstrated that collectively, rare *RAD51C* MS variants are enriched in familial BC, and consistent with protein-truncating variants, are more strongly associated with ER-negative and TN BC. Based on excess in cases and in silico predictions, this study has identified several potentially pathogenic variants, however, the definitive designation is challenging due to the low frequency among the population. Nevertheless, our data do exclude some variants as being moderate- to high-penetrance variants. For example, p.Gly264Ser has previously been reported in several small studies to be associated with ovarian cancer and/or BC^4–6^, which was consistent with functional assays showing this variant caused the partial reduction of RAD51C cellular function including cell survival, mitomycin C sensitivity and homologous recombination activity^5,9^. However, in the more highly powered BEACCON study, the p.Gly264Ser allele was detected at similar frequencies in cases and controls and was not associated with loss of the wild-type allele in BCs from carriers. In addition, the tumour from the homozygous p.Gly264Ser carrier did not show a high HRD score or a strong mutational signature 3 that are characteristic of *RAD51C*-null tumours, indicating that its HR pathway remained largely intact. The data strongly suggest that despite in vitro functional assays showing p.Gly264Ser reduces the activity of RAD51C, it is not associated with increased risk of BC. Taken together, our data conflict with the suggestion that this variant may be pathogenic and highlight the need for caution when extrapolating from the results of functional assays to clinical classification of variants.

A number of rare variants have previously been reported as likely pathogenic, including p.Gln143Arg^7,8^, p.Arg258His^7,9,10^, p.Cys135Tyr^7,8,15^, p.Ile144Thr^7,16^ and p.Val169Ala^4,5^. In this study, p.Gln143Arg was detected in 0.7% of cases (*n* = 4), including one TN BC and two with a family history of ovarian cancer, and 0.2% of controls (*n* = 3), consistent with the observed *RAD51C* phenotypes. Pedigree segregation of family 22 also supported that the variant p.Gln143Arg segregated with two subjects affected with ductal BC. While previously described as unlikely to be pathogenic^17^, p.Arg212His was detected in this study in two cases (0.03%) and no controls, and was predicted as deleterious by all five in silico tools. On the other hand, p.Val169Ala was identified in 12 control subjects, three-fold higher than the case frequency, making it unlikely to be a pathogenic variant. Among 12 tumours sequenced across eight germline variants, biallelic inactivation and high HRD scores were observed in ER-negative BCs and ovarian cancer of p.Ile144Thr, p.Arg212His and p.Arg258His carriers but not in tumours of p.Glu143Arg, p.Asp242Asn, p.Ile244Val and p.Leu262Val carriers. HRD was associated with tumour features including TN subtype, locus-specific LOH and mutational signature 3 that were more pronounced in carriers of candidate variants. A high proportion of mutational signature 3 was observed in tumours from p.Ile144Thr and p.Arg258His carriers, and generally in TN tumours, indicating that HR-deficiency is prominent among this group. Promoter hyper-methylation, which has been observed in *BRCA1/2* tumours^18^, appears unlikely to be an important mechanism for *RAD51C*, with no instances observed in the tumours examined. Although the number of tumours and family members sequenced for each variant was low, when combined with the case–control results, the data provided support for further investigation of those variants identified in this study as candidates by expansion or pooling of databases.

While this study generated evidence against the pathogenicity of p.Gly264Ser, there are several limitations to interpreting results for other variants. Despite a large sample size of ~20,000 subjects, the power of the study was limited in its capacity to identify and assess individual rare variants. For the variants examined here, most of which have a MAF of ~10^−5^, to securely identify an odds ratio of >2 would require a sample size of several million (~4.7 million total cases and controls by standard power calculation). Such numbers seem unachievable even with extensive international collaboration. The statistical power is further eroded by the fact that recent findings indicate that only the rarer TN subset of BC is attributable to *RAD51C*^1–3^. Given these limitations of case–control analyses, insights from tumour sequencing, including identifying a “second hit” and characteristic genome alterations, may offer the best avenue for validating or refuting a role for *RAD51C* MS variants in BC predisposition.

Evidence from this study supports an association of *RAD51C* MS variants with familial BC but due to their rarity, case–control results were not sufficiently powered to identify individual pathogenic variants. Tumour sequencing provided an additional tool to interrogate the in vivo consequences of candidate variants and robustly classified some variants as benign. Integrated analyses of case–control and tumour sequencing findings showed that the p.Gly264Ser variant is unlikely to be a moderate- to high-penetrance variant, despite in vitro assays showing partial functional impairment. These findings raise questions about the validity of functional assays as accurate predictors of variant pathogenicity. While further studies are required for rare variants, integration of case–control data with tumour sequencing provides a powerful strategy to clarify the role of *RAD51C* MS variants in BC predisposition.

## Methods

### Subject cohorts

The case cohort comprised of female index patients diagnosed with BC from 5,734 hereditary breast and ovarian cancer families identified from the ViP Study (combined Victorian and Tasmanian Familial Cancer Centres, Australia) and Pathology North (NSW Health Pathology, Newcastle, Australia). The cases were determined eligible for clinical genetic testing for hereditary BC predisposition genes based on personal and/or family history by a specialist Familial Cancer Clinic. All case subjects have been tested negative for *BRCA1*/*BRCA2* pathogenic variants prior to recruitment. The controls were 14,382 cancer-free female subjects from the Lifepool Study (http://www.lifepool.org/) in Victoria, Australia (BreastScreen Victoria). The average age of the first diagnosis in cases was 45.8 years (range, 17–85), while the average age of controls in this study was 64.4 years (range, 40–97), indicating a design that enriches for lifetime cancer-free controls. Family history of cancer was recorded for all cohort subjects by questionnaire or in-person interview. Cases ascertained through ViP study were provided with detailed pedigrees with breast and ovarian cancer family history verified against state cancer registries, and tumour pathology reports. This study was approved by the Human Research Ethics Committees at each participating ViP study recruitment centre and the Peter MacCallum Cancer Centre (Approval # 09/29). All participants provided informed consent for genetic analysis of their germline DNA (cases and controls) and tumour DNA (cases only).

### Targeted sequencing of germline DNA of cases and controls

The coding region and exon-intron boundaries (at least 10 bp of each intron) of *RAD51C* in germline DNA samples were amplified using a custom-designed HaloPlex Targeted Enrichment Assay panel (Agilent Technologies, Santa Clara, CA) according to the manufacturer’s protocol, and the libraries were sequenced on a HiSeq 2500 Genome Analyzer (Illumina, San Diego, CA) (100 or 150 bp paired-end reads). LoF variants were defined as stop-gained, frameshift or essential splice-site variants. MS variants were defined as non-synonymous single nucleotide variants.

### Sequencing of tumour DNA of *RAD51C* MS carriers

Tumour DNA was collected from cancer cells in formalin-fixed, paraffin-embedded slides by needle microdissection under the microscope. For targeted sequencing, all exons of *RAD51C* and 487 additional genes (including 27 BC driver genes) were amplified using a SureSelect XT Custom Panel (Agilent Technologies, Santa Clara, CA); for whole-exome sequencing, all exons were amplified using a SureSelect Human All Exon V8 Panel (Agilent Technologies, Santa Clara, CA). The libraries were sequenced on an NextSeq 500 Sequencing System (Illumina, San Diego, CA) (75 bp paired-end reads). Sanger sequencing was carried out using exon-specific primers (designed using Primer3^19^) and BigDye Terminator v3.1 kit (Thermo Fisher Scientific, Waltham, MA). Promoter hyper-methylation was determined by Sanger sequencing of bisulfite-converted tumour DNA using EpiTect Bisulfite Kit (Qiagen, Hilden, Germany).

### Identification of MS variants

Sequencing results were aligned to the g1 k x27 h19 reference genome using the Burrows-Wheeler Alignment tool^20^, SNP variant calling was carried out using GATK UnifiedGenotyper v2.4 (Broad Institute, Cambridge, MA), Platypus^21^ and Varscan^22^, and variants were annotated using the Ensembl Variant Effect Predictor^23^. Rare MS variants were identified in the canonical transcript by at least two variant callers, with sequencing quality ≥30, allele frequency ≥20% and MAF ≤ 0.005 for MS variants in non-Finnish European in gnomAD (Version 2.1, released 17 October 2018)^24^. Manual examination of BAM files and Sanger sequencing was carried out for ambiguous variants to remove sequencing artefacts. The positions of rare MS variants on the *RAD51C* gene were visualised using cBioPortal^25^. In silico tools, CADD^26^, REVEL^27^, Polyphen^28^, SIFT^29^ and Condel^30^, were used to predict the deleteriousness of an MS variant.

### Analysis of copy number alteration and homologous recombination deficiency (HRD) score

A genome-wide copy number plot was generated for each tumour using off-target reads via the copywriteR package in R studio^31^ and visualised using NEXUS Copy Number™ v8.0 (BioDiscovery Inc, El Segundo, CA, USA). Copy number alteration at the loci of *RAD51C* and other known HR genes on chromosome 17q, *BRCA1* and *RAD51D*, were determined. An HRD score was calculated for each tumour sample as a sum of the occurrence of telomeric allelic imbalances^32^, large-scale state transitions^33^ and HRD–LOH^34^.

### Sliding window analysis

MS variants were separated into each unique window of N amino acids, then Fisher’s exact test was performed using the counts of variants in the case and control samples. *P* values were then adjusted based on the null distribution estimated by randomising the sample labels of each variant and recalculating the optimal *p* value for each iteration.

### Mutational signature analysis

Rare somatic mutations were identified after filtering against germline variants, removing intron variants, sequencing read depth ≥20, allele frequency ≥10% and MAF ≤ 0.0001 for in non-Finnish European in gnomAD. As the number of somatic mutations was low in individual targeted panel sequenced samples, mutations were pooled into groups according to variant type and/or tumour pathology. Mutational signatures (COSMIC v2) were generated using the DeconstructSig package in R^35^.

### Statistical analysis

Odds ratios and Fisher’s exact test (two-sided) were examined for the case–control analysis, with a two-tailed *p* value of ≤0.05 designated as statistically significant, and confidence intervals were calculated using conditional Maximum Likelihood Estimate. All calculations were carried out using R-in built function in R 3.3.2^36^.

### Reporting summary

Further information on research design is available in the Nature Research Reporting Summary linked to this article.

## Supplementary information

Supplementary File

Reporting Summary

## Data Availability

All sequencing data are deposited to the European Genome-phenome Archive under accession number EGAD00001007025.

## Code availability

Standard R codes were used. Code requests should be addressed to I.G.C.
