# Supplementary material for: Integration of tumour sequencing and case–control data to assess pathogenicity of RAD51C missense variants in familial breast cancer
Source: NPJ Breast Cancer. 2022 Jan 17;8:10. doi: 10.1038/s41523-021-00373-y (PMC8763908; doi:10.1038/s41523-021-00373-y)
Supplement: Supplementary file 1 — Supplementary File [file 41523_2021_373_MOESM1_ESM.pdf]

Lim et. al. Supplementary Document

Supplementary Table 1: *RAD51C* rare missense variants detected in the BEACCON study of 5,734 familial breast cancer cases and 14,382 population controls, and their characteristics.

Supplementary Table 2: Details of tumours with high HRD scores (HRD ≥ 42) including hormone receptor status, tumour variant allelic status, somatic mutation of *TP53* and zygosity of HR genes located on chromosome 17, *BRCA1, RAD51C* and *RAD51D.*

Supplementary Figure 1: HRD score analysis on variant status and hormone receptor subtype across 20 breast tumours of germline variant carriers. Candidate variants include p.Glu143Arg, p.Ile144Thr, p.Arg212His, p.Asp242Asn, p.Ile244Val, p.Arg258His and p.Leu262Val.

Supplementary Figure 2. Mutational signatures from combined *RAD51C* MS samples using DeconstructSig (A) VUS (cases 10 to 19 and 21), (B) VUS-TN group (cases 10, 14, 16 and 19), and (C) VUS-ER+ group (cases 12, 15, 17, 18 and 21), (D) p.Gly264Ser variant (cases 1 to 9), (E) p.Gly264Ser-TN group (cases 1 to 5), (F) p.Gly264Ser-ER+ group (cases 7 to 9), (G) case 1 TN with germline homozygous p.Gly264Ser, (H) case 2 TN with LOH retaining p.Gly264Ser, (I) case 14 TN with LOH retaining p.Ile144Thr, (J) case 16 with LOH retaining p.Arg212His, (K) case 20 ovarian tumour with LOH retaining p.Arg258His, and (L) combined tumours with LOH in a LoF variant as reference[†].

Supplementary Figure 3: Pedigree analyses of families of (A) subjects 4 and 6, (B) subject 9, (C) subject 12, (D) subject 22, (E) subject 23, (F) subject 24, and (G) subject 25. Tumour hormone receptor status, lobular breast cancer and age of diagnosis are specified where available.

Supplementary Table 1: *RAD51C* rare missense variants detected in the BEACCON study of 5,734 familial breast cancer cases and 14,382 population controls, and their characteristics

	Frequency		MAF	*In silico* prediction					ClinVar	Interaction domain	Pathology or family phenotype			Literature reported
**HGVSp**	**case (homozygous) (%)**	**Control (homozygous) (%)**		**REVEL**	**CADD**	**Condel**	**PolyPhen**	**SIFT**			**TN (available)**	**Index OC**	**FHx OC**	
p.Thr5Met	1	1	4.00E-05	0.04	10	neu	neu	del	VUS	0				
**p.Gln11Arg**	0	1	1.00E-05	0.19	26	del	del	del	VUS	0				
**p.Lys26Glu**	0	1	<1.00E-05	0.24	28	del	del	del	.	0				
p.Lys26Arg	1	0	<1.00E-05	0.06	23	neu	neu	neu	.	0	0 (1)			
p.Ser29Phe	1	0	<1.00E-06	0.11	24	neu	neu	del	VUS	0	0 (1)			
**p.Ala30Val**	0	1	<1.00E-05	0.21	26	del	del	del	VUS	0				
p.Gln33Arg	0	1 (1)	<1.00E-07	0.04	23	neu	neu	neu	.	0				
p.Ile52Leu	0	1	<1.00E-08	0.04	22	neu	neu	neu	VUS	0				
p.Thr78Ile	1	1	<1.00E-09	0.06	21	neu	neu	neu	VUS	0	0 (1)			
**p.Lys84Asn**	1	0	1.00E-05	0.08	19	neu	neu	neu	VUS	1	0 (1)			
**p.Thr96Ser**	1	0	2.00E-05	0.11	18	neu	neu	neu	VUS	1				
p.Ala126Thr†	68 (1.19%)	133 (0.90%)	0.0054	0.1	22	neu	neu	neu	B	1	11 (33)	1	2	Successful correction of (MMC) hypersensitivity [3]; Benign [2]; no changes in protein interaction [7]
**p.Cys135Tyr**	1	1	1.00E-05	0.75	27	del	del	del	LP	1				Partially deleterious[1]
**p.Met136Ile**	1	0	2.00E-05	0.05	17	neu	neu	neu	VUS	1	1 (1)			

**p.Gln137Arg**	1	0	<1.00E-05	0.63	26	del	del	del	.	0	0 (1)			
**p.Gln143Arg**	4 (0.07%)	3 (0.02%)	8.00E-05	0.69	26	del	del	del	VUS	0	1 (3)		2	Partially deleterious[1] Pathogenic[2]
**p.Ile144Thr**	2	1	8.00E-05	0.31	26	del	del	del	VUS	0	1 (2)			
p.Ser163Gly	1	2	<1.00E-05	0.26	23	neu	neu	neu	VUS	0				
p.Val166Ala	1	0	2.00E-05	0.06	23	neu	neu	neu	VUS	0				
p.Val169Ala	2 (0.03%)	12 (0.08%)	0.0003	0.08	22	neu	neu	neu	VUS	0	0 (1)			Successful correction of (MMC) hypersensitivity [3]
**p.Ala175Thr**	1	1	4.00E-05	0.44	26	del	del	del	VUS	0				
**p.Cys176Arg**	0	1	<1.00E-05	0.33	26	del	del	del	.	0				Excess in case[3]
p.Gln178Pro	1	0	<1.00E-05	0.21	24	neu	neu	del	.	0			1	
p.His187Asp	0	1	<1.00E-05	0.04	15	neu	neu	neu	.	0				
p.Arg193Gln	0	1	3.00E-05	0.07	13	neu	neu	neu	VUS	0				
p.Ile208Val	1	1	3.00E-05	0.13	24	neu	neu	neu	VUS	0				
**p.Arg212His**	2	0	2.00E-05	0.64	28	del	del	del	VUS	0	0 (2)			Benign[4]
p.Arg214His	0	2	<1.00E-05	0.17	14	neu	neu	neu	VUS	0				
p.Ser231Thr	0	1	<1.00E-06	0.11	23	neu	neu	neu	.	0				
p.Glu232Asp	1	0	<1.00E-07	0.15	18	neu	neu	neu	VUS	0				
p.Ile240Leu	0	1	1.00E-05	0.25	23	neu	neu	del	VUS	0				
**p.Val241Met**	1	1	3.00E-05	0.52	25	del	del	del	VUS	0	0 (1)			
**p.Asp242Asn**	1	0	<1.00E-05	0.81	32	del	del	del	VUS	0	0 (1)			
p.Ile244Val	1	1	4.00E-05	0.16	20	neu	neu	neu	VUS	0	1 (1)			
**p.Arg249Cys**	0	1	1.00E-05	0.44	30	del	del	del	VUS	0				
**p.Arg249His**	0	1	<1.00E-05	0.44	30	del	del	del	VUS	0				
p.His250Tyr	0	1	3.00E-05	0.13	24	neu	neu	del	VUS	0				
p.Asp253Gly	0	1	1.00E-05	0.21	27	del	neu	del	VUS	0				
**p.Arg258Cys**	1	1	6.00E-05	0.53	28	del	del	del	VUS	0	0 (1)			

**p.Arg258His**	2 (0.03%)	4 (0.03%)	4.00E-05	0.5	32	del	del	del		0	0 (2)	1		Deleterious via functional[5] Pathogenic[2], Hypomorphic[6]
**p.Arg260Trp**	0	1	<1.00E-05	0.6	29	del	del	del	VUS	0				
**p.Leu262Val**	1	1	0.0001	0.32	22	del	del	del	VUS	0	0 (1)		1	
p.Gly264Ser	30 (1) (0.52%)	78 (0.53%)	0.0034	0.2	23	neu	neu	neu		0	10 (14)		1	Pathogenic in small sample size[3, 8, 9]; reduced cell survival [3], Hypomorphic[6]
**p.Met286Thr**	0	1	<1.0E-05	0.42	26	del	del	del	.	0				
p.Met286Val	0	1	<1.00E-05	0.14	20	neu	neu	neu	VUS	0				
**p.Arg312Gln**	0	1	3.00E-05	0.47	32	del	del	del	VUS	0				
p.Thr336Pro	1	1	2.00E-05	0.21	23	neu	neu	del	VUS	0				
p.Val350Ile	1	1	<1.00E-05	0.02	16	neu	neu	neu	.	0	0 (1)			
p.Ala354Val	0	1	2.00E-05	0.04	12	neu	neu	neu	VUS	0				
p.Gln358Glu	0	1	<1.00E-05	0.06	0	neu	neu	neu	.	0				
p.Ser364Gly	1	0	2.00E-05	0.03	15	neu	neu	del	VUS	0				
p.Arg370Gln	0	1	2.00E-05	0.15	23	neu	neu	del	VUS	0				

Variants of interest that fulfil one or more of following criteria are in bold: (1) ≥4 *in-silico* tools predicted as pathogenic, (2) tumour or family phenotype of interest, (3) has a case-control excess, (4) located in interaction domain or (5) previously reported as pathogenic. Cut-offs of CADD≥25 and Revel≥0.5 are used. GnomAD_AF_NFE: GnomAD Database Allele Frequency Non-Finnish European; del: deleterious; neu: neutral; LP: likely pathogenic; VUS: variant of unknown significance; LB: likely benign; OC: ovarian cancer; FHx: 1-2 deg family history. ClinVar database was accessed in March 2020. Four likely TN cases (ER-/PR-/HER2nr) are included in this table.

†p.Ala126Thr is a known benign variant included as a reference

Supplementary Table 2: Details of tumours with high HRD scores (HRD ≥ 42) including hormone receptor status, tumour variant allelic status, somatic mutation of *TP53* and zygosity of HR genes† located on chromosome 17, *BRCA1, RAD51C* and *RAD51D*

**Sample**	***RAD51C* Variant**	**Hormone receptor/ HER2 status**	**Tumour Variant Allelic State**	**HRD score**	**Somatic Mutation**	**Zygosity**		
					TP53	***BRCA1***	***RAD51C***	***RAD51D***
3	p.Gly264Ser	TN	wt/-	122	mutant	na	hemi	na
11	p.Glu143Arg	TN	wt/-	67	mutant	hemi	hemi	hemi
14	p.Ile144Thr	TN	mut/-	78	mutant	hemi	hemi	hemi
16	p.Arg212His	ER-/HER2+	mut/-	47	mutant	hemi	hemi	hemi
18	p.Asp242Asn	ER+/HER2+	wt/-	49	WT	hemi	hemi	hemi
19	p.Ile244Val	TN	wt/-	78	mutant	na	hemi	hemi
20	p.Arg258His	OvCa	mut/-	70	mutant	hemi	hemi	hemi

‘na’- unreadable due to poor quality
‘Hemi’ – hemizygous
†Apart from *RAD51C*, no mutations were found in other known BC predisposing genes.

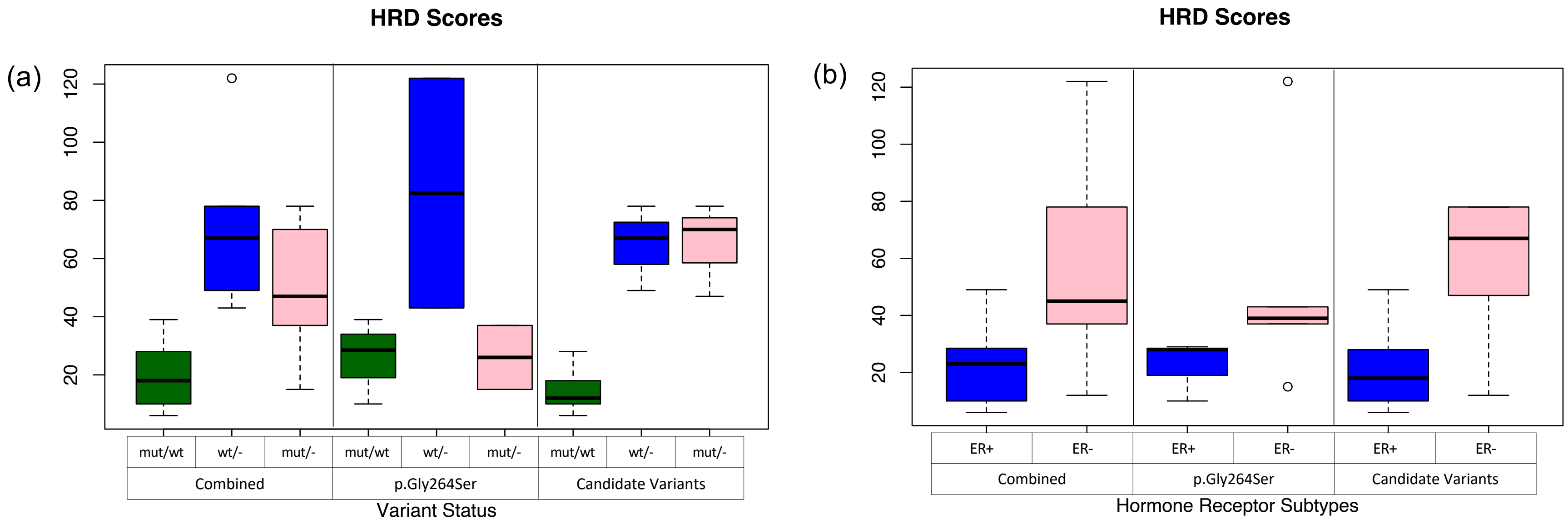

Supplementary Figure 1: HRD score analysis on variant status, hormone receptor subtype and *TP53* somatic mutation across 20 breast tumours of germline variant carriers. Candidate variants include p.Glu143Arg, p.Ile144Thr, p.Arg212His, p.Asp242Asn, p.Ile244Val, p.Arg258His and p.Leu262Val.

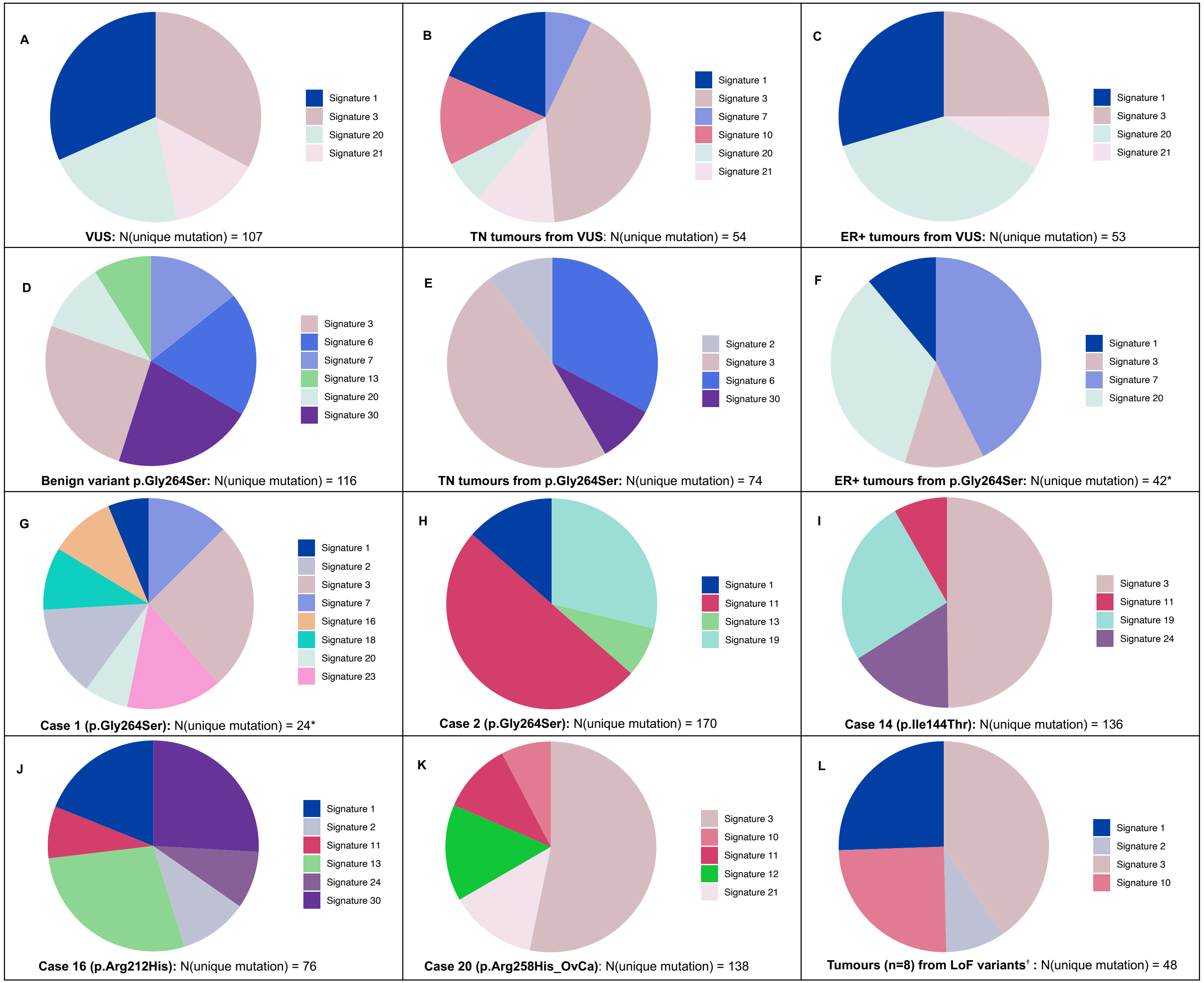
A
Signature 1
Signature 3
Signature 20
Signature 21
VUS: N(unique mutation) = 107
B
Signature 1
Signature 3
Signature 7
Signature 10
Signature 20
Signature 21
TN tumours from VUS: N(unique mutation) = 54
C
Signature 1
Signature 3
Signature 20
Signature 21
ER+ tumours from VUS: N(unique mutation) = 53
D
Signature 3
Signature 6
Signature 7
Signature 13
Signature 20
Signature 30
Benign variant p.Gly264Ser: N(unique mutation) = 116
E
Signature 2
Signature 3
Signature 6
Signature 30
TN tumours from p.Gly264Ser: N(unique mutation) = 74
F
Signature 1
Signature 3
Signature 7
Signature 20
ER+ tumours from p.Gly264Ser: N(unique mutation) = 42*
G
Signature 1
Signature 2
Signature 3
Signature 7
Signature 16
Signature 18
Signature 20
Signature 23
Case 1 (p.Gly264Ser): N(unique mutation) = 24*
H
Signature 1
Signature 11
Signature 13
Signature 19
Case 2 (p.Gly264Ser): N(unique mutation) = 170
I
Signature 3
Signature 11
Signature 19
Signature 24
Case 14 (p.Ile144Thr): N(unique mutation) = 136
J
Signature 1
Signature 2
Signature 11
Signature 13
Signature 24
Signature 30
Case 16 (p.Arg212His): N(unique mutation) = 76
K
Signature 3
Signature 10
Signature 11
Signature 12
Signature 21
Case 20 (p.Arg258His_OvCa): N(unique mutation) = 138
L
Signature 1
Signature 2
Signature 3
Signature 10
Tumours (n=8) from LoF variants† : N(unique mutation) = 48

Supplementary Figure 2. Mutational signatures from combined *RAD51C* MS samples using DeconstructSig (A) VUS (cases 10 to 19 and 21), (B) VUS-TN group (cases 10, 14, 16 and 19), and (C) VUS-ER+ group (cases 12, 15, 17, 18 and 21), (D) p.Gly264Ser variant (cases 1 to 9), (E) p.Gly264Ser-TN group (cases 1 to 5), (F) p.Gly264Ser-ER+ group (cases 7 to 9), (G) case 1 TN with germline homozygous p.Gly264Ser, (H) case 2 TN with LOH retaining p.Gly264Ser, (I) case 14 TN with LOH retaining p.Ile144Thr, (J) case 16 with LOH retaining p.Arg212His, (K) case 20 ovarian tumour with LOH retaining p.Arg258His, and (L) combined tumours with LOH in a LoF variant as reference†

Variant criteria: Consequence_rank≤6; GnomAD_AF_NFE≤0.0001; AlleleFreq≥0.1, ReadDepth≥20. VUS: Variants of unknown significance; TN: triple-negative; ER+: ER-positive. Combined mutational signatures were generated using large panel sequencing data, individual mutational signatures were generated using whole exome sequencing data.

†Previously published(1)
*number of somatic mutation input is less than n=50

(A)

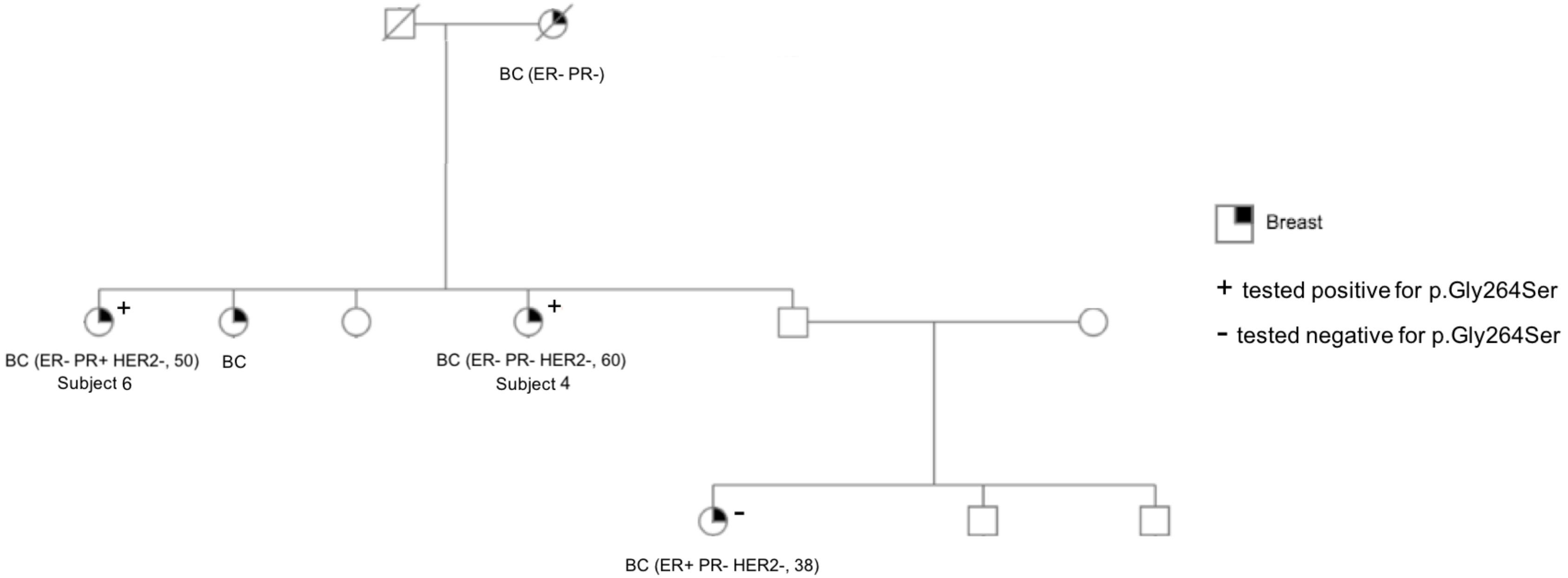
BC (ER- PR-)
+
BC (ER- PR+ HER2-, 50)
Subject 6
BC
+
BC (ER- PR- HER2-, 60)
Subject 4
-
BC (ER+ PR- HER2-, 38)
Breast
+ tested positive for p.Gly264Ser
- tested negative for p.Gly264Ser

(B)

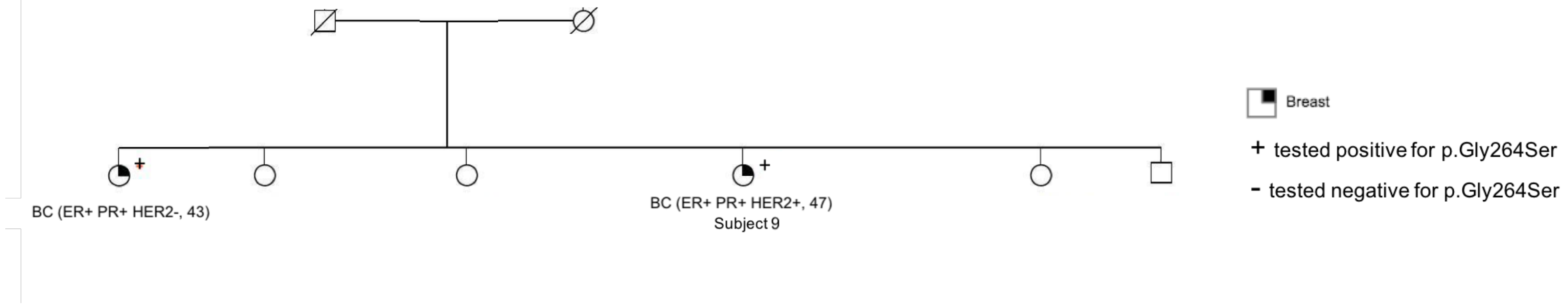
+
+
BC (ER+ PR+ HER2-, 43)
BC (ER+ PR+ HER2+, 47)
Subject 9
Breast
+ tested positive for p.Gly264Ser
- tested negative for p.Gly264Ser

(C)

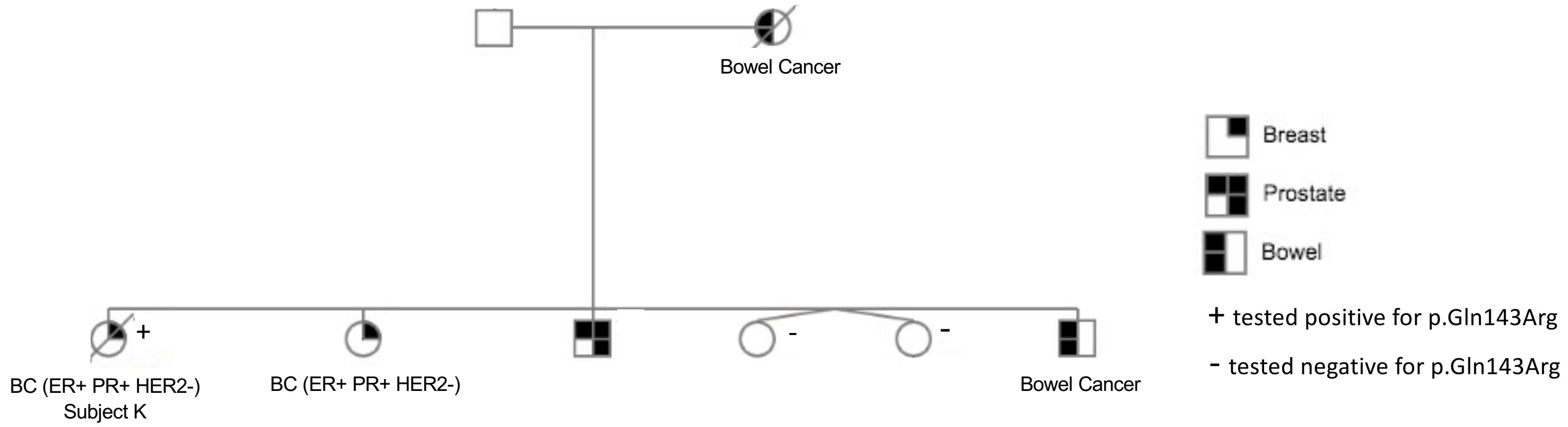
Bowel Cancer
Breast
Prostate
Bowel
+
-
-
BC (ER+ PR+ HER2-)
Subject K
BC (ER+ PR+ HER2-)
Bowel Cancer
+ tested positive for p.Gln143Arg
- tested negative for p.Gln143Arg

(D)

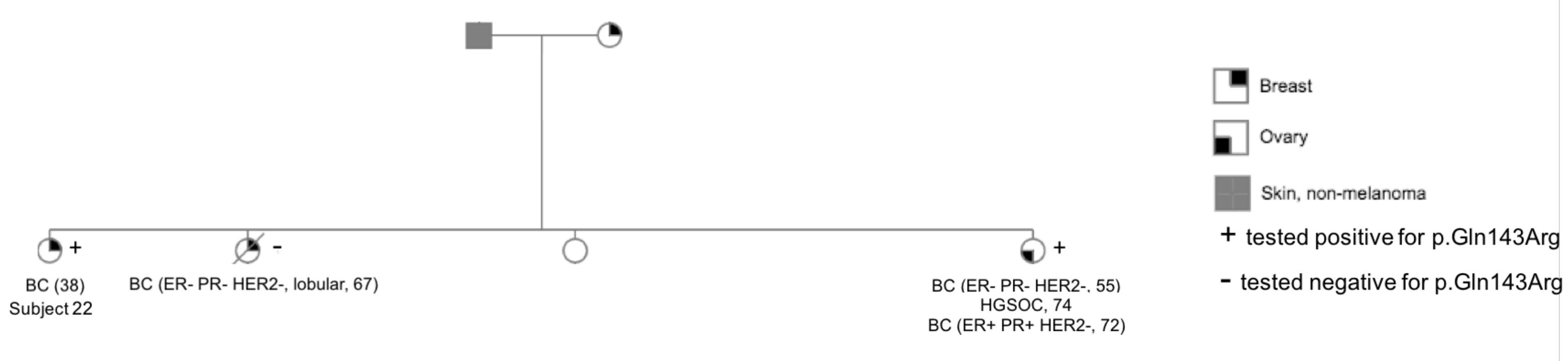
+
-
+
BC (38)
Subject 22
BC (ER- PR- HER2-, lobular, 67)
BC (ER- PR- HER2-, 55)
HGSOC, 74
BC (ER+ PR+ HER2-, 72)
Breast
Ovary
Skin, non-melanoma
+ tested positive for p.Gln143Arg
- tested negative for p.Gln143Arg

(E)

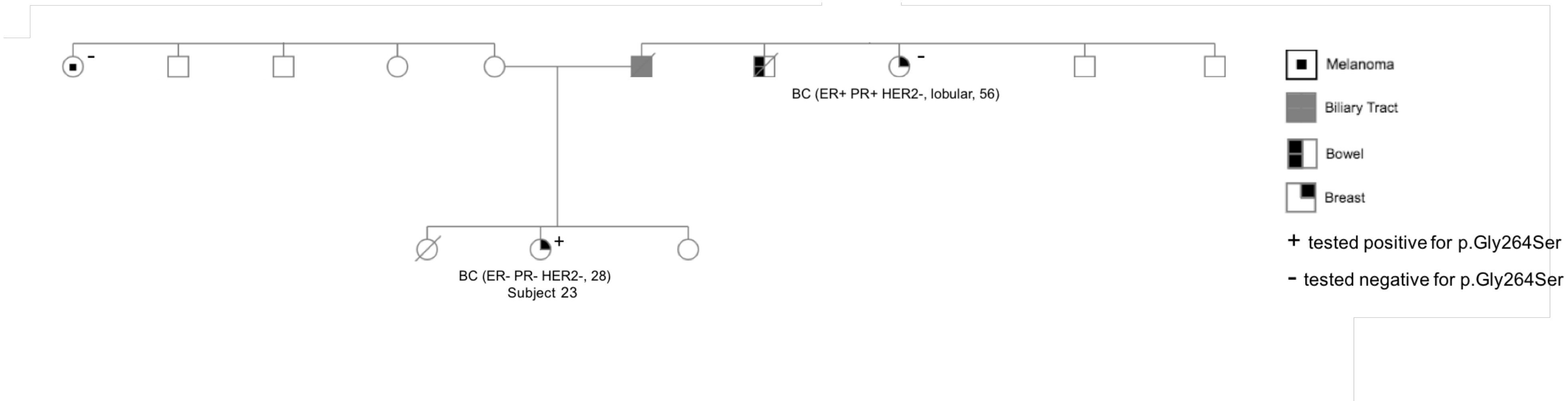
-
-
+
BC (ER+ PR+ HER2-, lobular, 56)
BC (ER- PR- HER2-, 28)
Subject 23
Melanoma
Biliary Tract
Bowel
Breast
+ tested positive for p.Gly264Ser
- tested negative for p.Gly264Ser

(F)

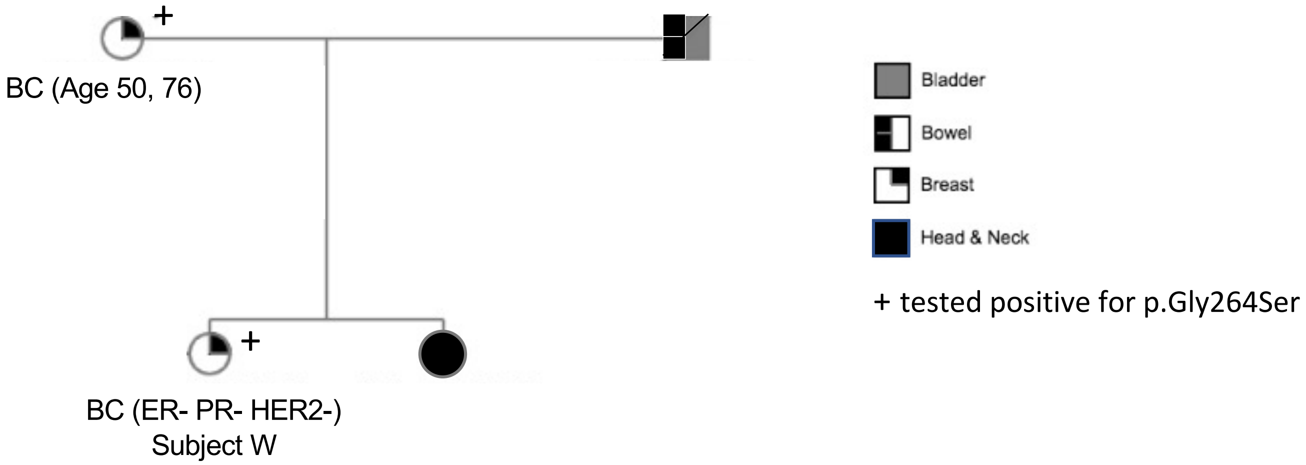
+
BC (Age 50, 76)
+
BC (ER- PR- HER2-)
Subject W
Bladder
Bowel
Breast
Head & Neck
+ tested positive for p.Gly264Ser

(G)

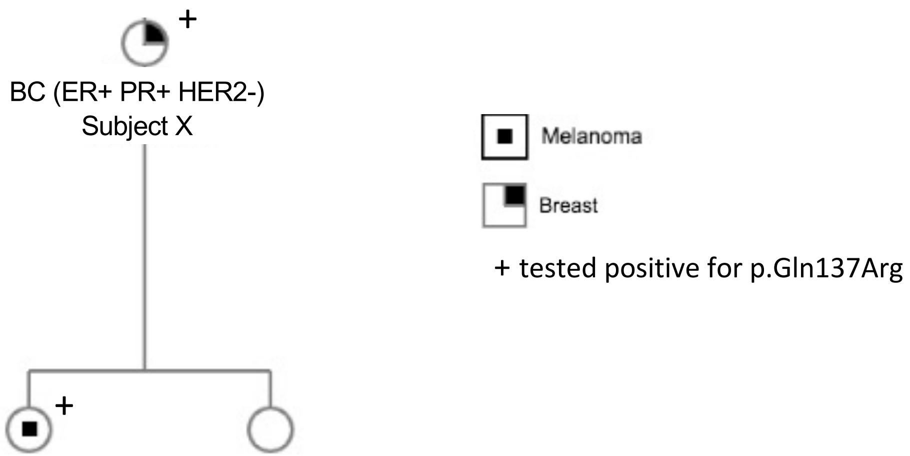
+
BC (ER+ PR+ HER2-)
Subject X
+
Melanoma
Breast
+ tested positive for p.Gln137Arg

Supplementary Figure 3: Pedigree analyses of families of (A) subjects 4 and 6, (B) subject 9, (C) subject 12, (D) subject 22, (E) subject 23, (F) subject 24, and (G) subject 25. Tumour hormone receptor status, lobular breast cancer and age of diagnosis are specified where available.

References

1. Li N, McInerny S, Zethoven M, Cheasley D, Lim BWX, Rowley SM, et al. Combined tumor sequencing and case/control analyses of *RAD51C* in breast cancer. Journal of the National Cancer Institute. 2019.
